# A DOUBLE-MASKED, RANDOMIZED, SHAM-CONTROLLED, SINGLE-CENTER STUDY WITH PHOTOBIOMODULATION FOR THE TREATMENT OF DRY AGE-RELATED MACULAR DEGENERATION

**DOI:** 10.1097/IAE.0000000000002632

**Published:** 2019-08-09

**Authors:** Samuel N. Markowitz, Robert G. Devenyi, Marion R. Munk, Cindy L. Croissant, Stephanie E. Tedford, Rene Rückert, Michael G. Walker, Beatriz E. Patino, Lina Chen, Monica Nido, Clark E. Tedford

**Affiliations:** * Department of Ophthalmology, University of Toronto, Toronto, Ontario, Canada;; † Department of Ophthalmology, University Health Network, Toronto, Ontario, Canada;; ‡ Department of Ophthalmology, Inselspital University Hospital Bern, Bern, Switzerland;; § LumiThera Inc, Poulsbo, Washington;; ¶ Eyegnos Consulting, Bern, Switzerland; and; ** Walker Bioscience, Carlsbad, California.

**Keywords:** dry age-related macular degeneration, drusen, light-emitting diode, low-level light therapy, photobiomodulation, vision loss, best-corrected visual acuity, mitochondria, contrast sensitivity

## Abstract

**Purpose:**

The LIGHTSITE I study investigated the efficacy and safety of photobiomodulation (PBM) treatment in subjects with dry age-related macular degeneration.

**Methods:**

Thirty subjects (46 eyes) were treated with the Valeda Light Delivery System, wherein subjects underwent two series of treatments (3× per week for 3–4 weeks) over 1 year. Outcome measures included best-corrected visual acuity, contrast sensitivity, microperimetry, central drusen volume and drusen thickness, and quality of life assessments.

**Results:**

Photobiomodulation-treated subjects showed a best-corrected visual acuity mean letter score gain of 4 letters immediately after each treatment series at Month 1 (M1) and Month 7 (M7). Approximately 50% of PBM-treated subjects showed improvement of ≥5 letters versus 13.6% in sham-treated subjects at M1. High responding subjects (≥5-letter improvement) in the PBM-treated group showed a gain of 8 letters after initial treatment (*P* < 0.01) and exhibited earlier stages of age-related macular degeneration disease. Statistically significant improvements in contrast sensitivity, central drusen volume, central drusen thickness, and quality of life were observed (*P* < 0.05). No device-related adverse events were reported.

**Conclusion:**

Photobiomodulation treatment statistically improved clinical and anatomical outcomes with more robust benefits observed in subjects with earlier stages of dry age-related macular degeneration. Repeated PBM treatments are necessary to maintain benefits. These pilot findings support previous reports and suggest the utility of PBM as a safe and effective therapy in subjects with dry age-related macular degeneration.

## Abstract 2

Photobiomodulation treatment in a randomized, sham-controlled, single-center, double-masked study conducted in 46 eyes of patients with dry age-related macular degeneration significantly improved functional (visual acuity and contrast sensitivity) and anatomical (drusen volume and central drusen thickness) outcomes. Patient improvements were reported in the VFQ-25 assessing activities of daily living.

Age-related macular degeneration (AMD) is a retinal disease that results in irreversible, severe loss of vision, including legal blindness. Disease progression inevitably leads to significant visual dysfunction and serious compromises in quality of life (QoL). The prevalence of AMD is projected to affect 196 million by the year 2020 with an expected growth rate to 288 million in 2040.^1^

Progression of AMD is characterized by accumulation of membranous debris, lipofuscin, and extracellular material and complement deposition. The advanced late-stage dry form of AMD, which accounts for 80% to 90% of the cases, is characterized by retinal pigment epithelium (RPE) and outer retinal atrophy, whereas only 10% to 20% develop the exudative, wet late-stage form, with choroidal neovascularization (CNV) as a hallmark of respective disease.^2^ Contributing factors to RPE cell degeneration include mitochondrial dysfunction, oxidative stress, inflammation, and genetic disposition.^3^

Treatment is available for wet AMD through periodic intravitreal injections of anti–vascular endothelial growth factor compounds. The more frequent dry form of AMD has limited treatment options available other than lifestyle changes and the use of vitamin supplements, demonstrating a significant unmet clinical need for alternate treatment plans for an expanding population base.^4,5^

The use of photobiomodulation (PBM), previously termed low-level light therapy, involves targeted use of selected wavelengths of visible light to near infrared (NIR) light (500–1,000 nm) produced by a laser or a noncoherent light source such as light-emitting diodes. Photobiomodulation can be applied to selected tissues to produce beneficial cellular effects leading to improved outcomes at the cellular, systemic, and clinical level in a wide range of disease states.^6–9^ The driving mechanism behind these benefits suggests that the mitochondrial enzyme cytochrome C oxidase is a key photoacceptor of light in the far red to NIR spectral range.^10–13^ The beneficial effects of PBM are linked to increases in mitochondrial energy generation through ATP, replication, density, and activity and increases in RNA and protein synthesis.^14^

The use of PBM in ocular diseases and disorders has been studied in both preclinical and clinical settings. In animal models of ocular injury, PBM has reduced damage or symptoms associated with methanol-toxicity, laser burn, complement factor H knockout inflammatory, bright light damage, retinitis pigmentosa, and diabetic retinopathy.^15–19^ Limited clinical studies show high potential for the use of PBM in the ocular field. Ivandic and Ivandic^20^ have shown clinical improvements in patients with amblyopia, retinitis pigmentosa, and AMD after treatment with PBM.^20–22^ In subjects with AMD, treatment with a laser diode aimed at the macular area improved visual acuity in both subjects with dry and wet AMD. No changes in visual acuity were seen in the control group, and there were no reports of any adverse effects among PBM-treated patients.^20^ Most recently, the Toronto and Oak Ridge PBM Studies for Dry Age-Related Macular Degeneration (TORPA I and II) presented evidence for clinical (improvements in best-corrected visual acuity [BCVA] and contrast sensitivity [CS]) and anatomical (reductions in drusen volume) benefits after PBM in patients with dry AMD.^23,24^ These positive clinical findings coupled with the known mitochondrial-based mode of action of PBM and the underlying pathology associated with AMD highly suggest that PBM treatment could have a therapeutic role in dry AMD, a condition that is characterized by mitochondrial dysfunction, oxidative stress, and inflammation within the RPE cell layer.

The current study further investigates the effects of PBM treatment on subjects with dry AMD in a double-masked, randomized, sham-controlled, parallel group, single-center prospective design. The primary goal of this study was to evaluate the efficacy and safety of PBM in subjects with dry AMD using the Valeda Light Delivery System, specifically designed for the ophthalmological use of PBM.

## Methods

### Subject Selection and Setting

Subjects were eligible for trial enrollment if they had dry AMD and were in Age-Related Eye Disease Study (AREDS) categories 2 to 4 with BCVA scores as determined by the Early Treatment Diabetic Retinopathy Study (ETDRS) Visual Acuity chart with a letter score between 50 and 85 (Snellen equivalent of 20/40 to 20/200). Subjects were excluded from enrollment with previous/active wet AMD, with a history of epilepsy, with cognitive impairment, other significant retinal disease, or other significant disease. Subjects could use AREDS vitamin supplementation; however, no change in supplements 1 month before the study and during the study trial was allowed. A total of 40 subjects were screened for the study, of which 30 subjects were randomized into the study. Both eyes were included if inclusion criteria were met in both eyes. Therefore, an adapted AREDS classification was used, as each eye was individually assessed for the presence of center involving geographic atrophy (GA). Thus, the fellow eye was not automatically deemed AREDS Category 4 if the other eye showed center involving GA. A total of 46 eyes were treated and analyzed.

This study took place at a single site located in Toronto, ON, Canada. This study was conducted in compliance with the protocol, Good Clinical Practice guidelines, Health Canada regulatory requirements, and all other applicable regulatory requirements. This study was performed in adherence to the guidelines of the Declaration of Helsinki.

### Study Design

**Fig. 1.**
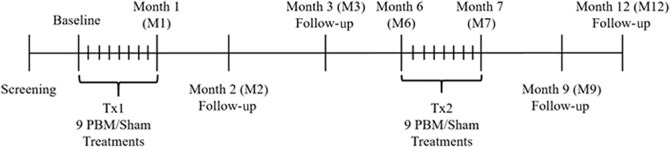
Diagram illustrating the LIGHTSITE I clinical study visit design. Subjects who met the inclusion/exclusion criteria at the screening and baseline visits were enrolled into the study. Subjects received two series of PBM treatments (Tx1 and Tx2) with a total of 9 treatment sessions per series distributed over 3 weeks to 4 weeks. Subjects underwent assessments at subsequent follow-up visits. The study comprised of 24 visits over the course of 1 year.

**Fig. 2.**
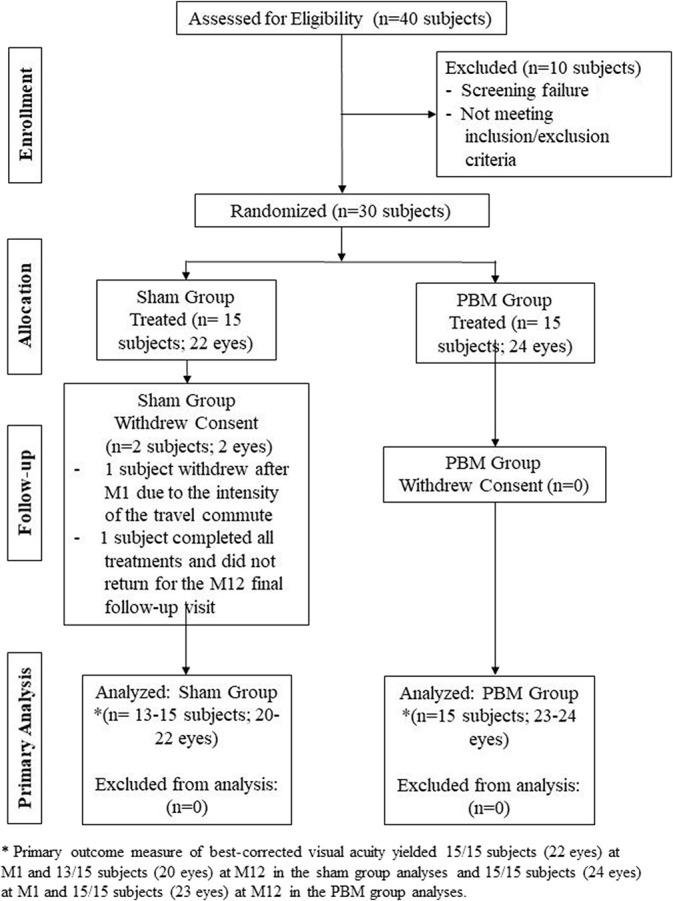
LIGHTSITE I consort diagram. Study subject progress through each study phase.

This prospective study was conducted in a double-masked, randomized, sham-controlled, parallel group format at a single clinical site. Data were collected during 24 visits over the course of the 1-year study (Figure 1). Subjects with dry AMD who met the inclusion criteria, had none of the exclusion criteria, and gave their written informed consent underwent sham or PBM treatment randomized at a 1:1 ratio. Subjects underwent two treatment series during the course of the study which consisted of sham or PBM treatments three times per week for three weeks, initiated at baseline and repeated at 6 months with subsequent follow-up visits after each treatment series. A consort diagram is provided in Figure 2. Subjects and study staff were masked to the treatment.

### Evaluated Parameters

Subjects were assessed for BCVA using the ETDRS charts (Precision Vision) and CS at 1.5, 3, 6, 12, and 18 cycles per degree (Levels A–E) (FACT, Stereo Vision Optec 6500) before and after treatment. Quality of life was assessed using the visual function questionnaire-25 (VFQ-25), and retinal sensitivity was recorded using microperimetry C10-2 grid with 68 tested points (MAIA; Centervue).

All subjects were assessed with 20 × 20 high-speed SD-OCT volume scans (Spectralis OCT; Heidelberg Engineering, Heidelberg, Germany) consisting of 49 section scans each (118 *µ*m distance between each scan, 9 frames averaged) and with 2 central (one horizontal and one vertical) 30° line scans 36 times averaged at baseline and at selected visits for anatomical changes. Fundus autofluorescence (FAF) imaging with 488-nm wavelength (Spectralis OCT; Heidelberg Engineering) was performed at the same visits. Subsequent spectral domain optical coherence tomography (SD-OCT) scans were performed using the TruTrack follow-up function to allow for exact comparison of retina and drusen volume.

An independent, masked imaging expert reviewed OCT and FAF images to determine dry AMD etiology and confirm inclusion/exclusion criterion. SD-OCTs and FAF were analyzed for following parameters: aligned mean central retinal thickness, aligned mean retinal volume, GA lesion area, and aligned drusen volume. The presence of reticular pseudodrusen, refractile drusen, incomplete and complete outer retinal atrophy, incomplete and complete RPE and outer retinal atrophy (iRORA and cRORA [corresponds to GA]), evidence of a CNV, (pseudo)vitelliform lesions and irregularity/disruption of the external limiting membrane, ellipsoid zone, and interdigitation zone were assessed based on a predefined grading protocol.^25^

The size and growth of GA were quantitatively assessed by 488-nm FAF, using the Region Finder Analyser (Region Finder Software Heidelberg Engineering; Heidelberg Engineering).^26^ The area of homogenous hypoautofluorescence on the FAF images at baseline and follow-up images was measured and quantified by one independent masked grader.^26^ The absolute GA lesion area was used to evaluate growth rate independent of initial lesion size.^27^

### Photobiomodulation Treatment

**Fig. 3.**
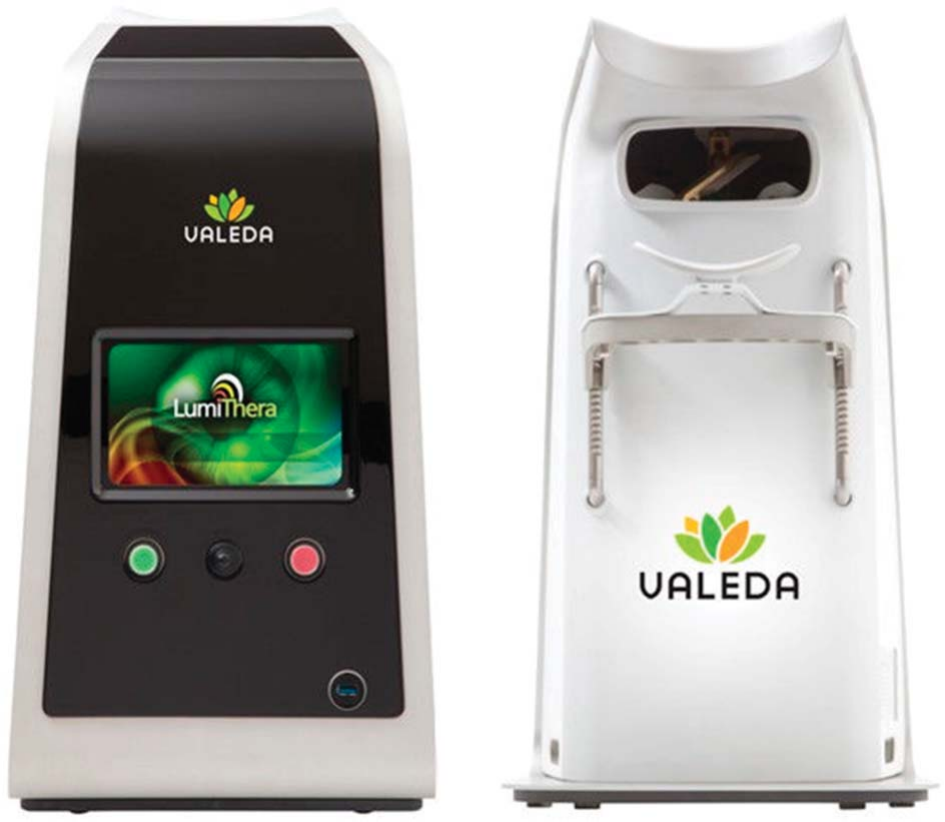
The Lumithera Valeda Light Delivery System. Illustration of the Valeda front (left) and backside (right).

Subjects were treated with the LumiThera Valeda Light Delivery System (Figure 3) which delivers three distinct wavelengths in the yellow (590 nm), red (660 nm), and NIR (850 nm) range. The Valeda Light Delivery System parameters are presented in Table 1. Masking of the study was accomplished through the use of the sham (placebo) treatment which delivered a noneffective dose of the selected wavelengths. The sham mode delivered an approximate 100x reduction in treatment fluence compared with the PBM mode. The integrity of the masking of the treatment modalities was further ensured through the inherent design of the Valeda instrumentation. The performance/output of all visible and audible indicators, including the graphic user interface, was identical for both treatment modalities (i.e., other than the emission of visible and NIR PBM, the behavior of the system was identical for both the sham and PBM treatment). The Valeda device operator was further masked by operating the instrument under a cloth shield to prevent any accidental viewing of incidental light during the treatments. The Valeda Light Delivery System is an investigational device. The Valeda System is CE-marked but not approved for use by the FDA or Health Canada.

**Table 1.**
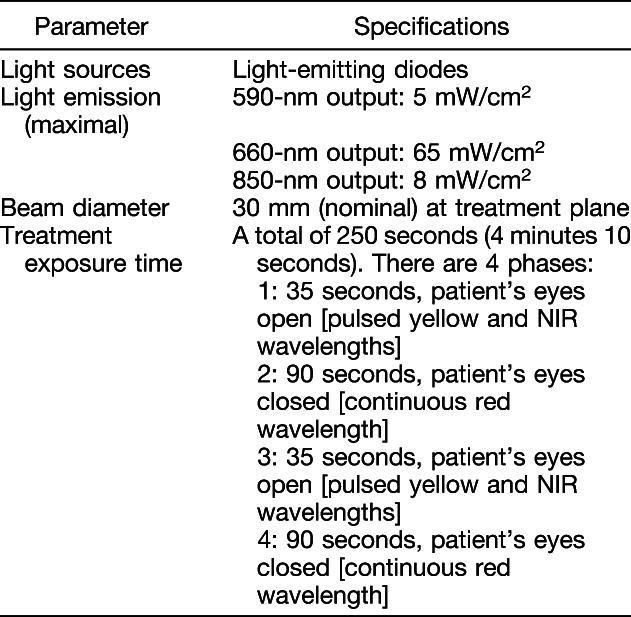
Valeda Light Delivery System Specifications

### Statistical Analyses

Statistical analyses were performed using R version 3.0 or higher (R: The R Project for Statistical Computing; https://www.r-project.org/). All analyses are based on individual eyes, rather than individual subjects, unless otherwise indicated. Linear mixed-effects (LME) analyses were performed using the R package NLME. Graphs were generated using the R package ggplot2. Best-corrected visual acuity, CS, and microperimetry comparisons were analyzed with a LME two-level hierarchical model, with eyes nested within subject, to account for correlation between eyes, with treatment group as a fixed effect and subject as a random effect. Changes before and after PBM within the PBM group and before and after sham within the sham group were analyzed using a Wilcoxon signed-rank test for paired data (significance set at *P* < 0.05). A Wilcoxon rank-sum (Mann–Whitney *U*) test was used to compare the difference between PBM-treated and sham-treated subjects in changes at selected intervals. The nonparametric (Wilcoxon) tests were not adjusted for correlation between eyes within subject. VFQ-25 analysis used a linear regression model. Fisher's exact test was used to analyze AREDS category distribution between treatment groups. Two-sided *P* values less than 0.05 were considered statistically significant. Adjustments for multiple comparisons were not performed. Analyses used the Intent to Treat (ITT) population, unless otherwise specified. All 30 subjects and 46 eyes randomized are included in the ITT population and the ITT analyses. Linear mixed-effects models were used to allow for the possibility of missing values at particular time points in ITT analyses.

## Results

The LIGHTSITE I study evaluated 30 subjects for a total of 46 qualifying eyes. The mean age for all subjects was 76 years (±8.3). A higher number of women (60%) than men (40%) were included. The median duration of dry AMD was 7.8 years (±7.6) since diagnosis.

The majority of subjects had intermediate to advanced stage dry AMD as categorized by high prevalence of subjects with AREDS categories 3 (30.4%) and 4 (67.4%) (Table 2). The majority of eyes had GA (73.9%). In the sham group, 52.9% of eyes that were categorized as AREDS Category 4 with central 1.0 mm involving GA also had foveola involvement. In the PBM group, 78.5% of eyes that were categorized as AREDS 4 with central 1.0 mm involving GA also had central foveola involvement. No statistical differences between the sham and PBM treatment groups were seen in the distribution of AREDS categories (Fisher's exact test, *P* = 0.27). In total, almost half (20/46 eyes, 43.5%) of all eyes in this study were categorized as AREDS 4 with foveola involving GA.

**Table 2.**
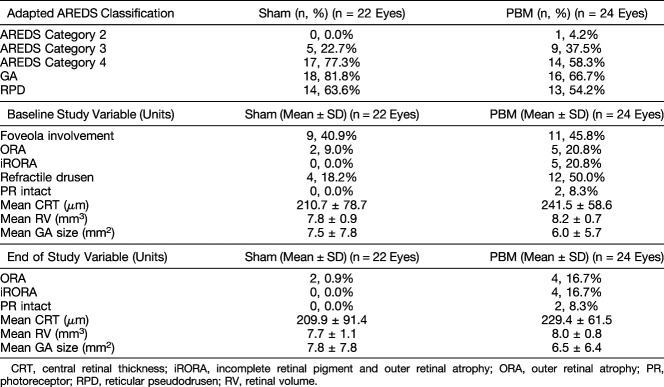
Baseline Disease Distribution According to Age-Related Eye Disease Study Classification and Study Variables

### Best-Corrected Visual Acuity Assessment

**Fig. 4.**
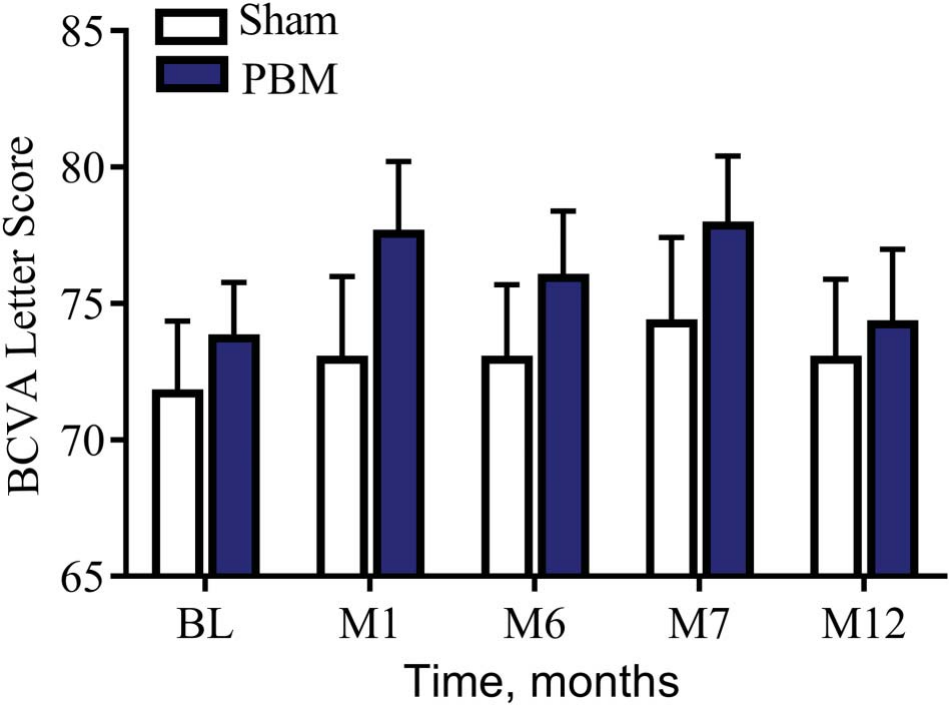
The effect of PBM on ETDRS BCVA letter score over-time. As a group, PBM-treated subjects showed an improvement in ∼4 letters immediately after treatment (M1 and M7) which diminished over time demonstrating the need for repeated treatment to maintain clinical benefits.

Sham- and PBM-treated subjects had similar baseline BCVA mean letter scores (sham, 71.9 ± 2.5; PBM, 73.8 ± 1.9). In the ITT analysis, after the initial treatment series at M1, sham subjects showed no significant change from BL with a one-letter improvement, whereas PBM-treated subjects showed an increase in BCVA of ∼4 letters to 77.7 ± 2.5 letters (Table 3). Photobiomodulation treatment effects on BCVA appeared to start to diminish at the M6 time point (76.1 ± 2.3 letters) just before retreatment. After the second series of PBM treatments at M7, PBM-treated subjects' mean BCVA improved to a letter score of 78 ± 2.4. The PBM benefits diminished again by M12 and returned to approximate prestudy BL BCVA levels (74.2 ± 2.6 letters) (Figure 4). High variability of BCVA changes across subjects supported additional analysis to further understand the subjects' individual responses.

**Table 3.**
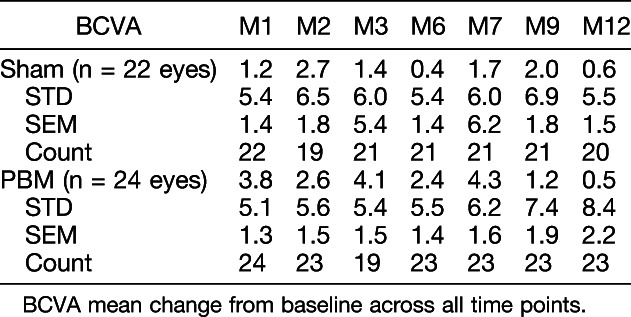
Best-Corrected Visual Acuity Outcomes Throughout 12-Month Time Course

**Fig. 5.**
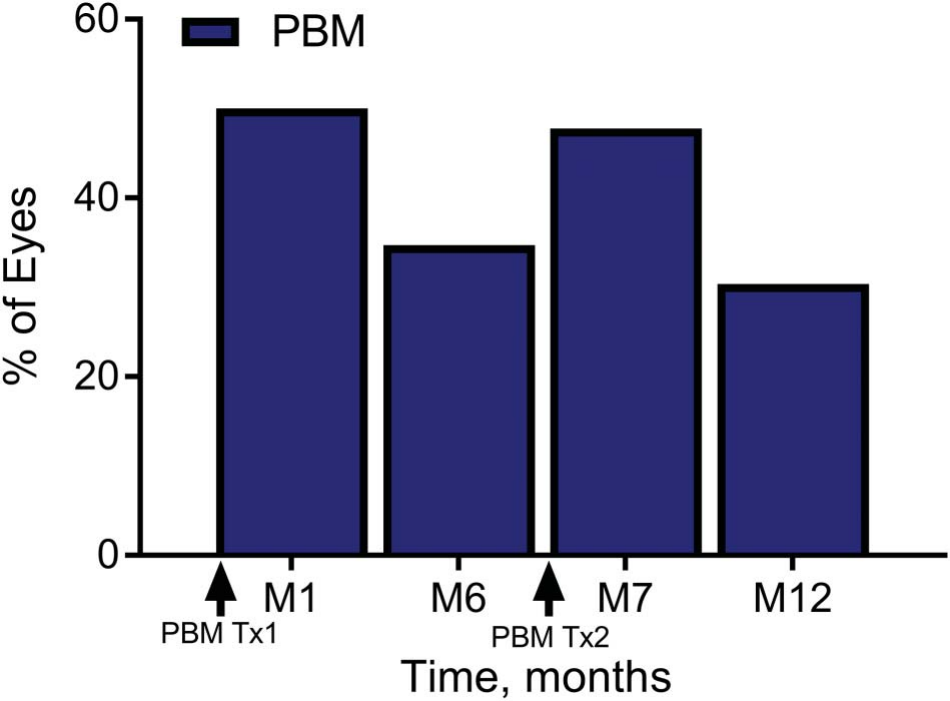
The percentage of PBM-treated subjects with a ≥5 letter improvement on ETDRS visual acuity from baseline over time. After each treatment series (M1 and M7), almost one half of all PBM-treated eyes showed ≥5 letter gain (50% of eyes at M1, 46% of eyes at M7). This effect was reduced over time until the next treatment series was initiated. Photobiomodulation-treated eyes that showed ≥5 letter gain were typically in earlier stages of the disease and did not have significant GA with foveola involvement.

Subsequent stratified analysis of the benefits of PBM treatment demonstrated that 50% of PBM-treated subjects showed improvement of 5 or more letters (one-line improvement or better, Figure 5) compared with only 13.6% of sham-treated subjects at M1. Photobiomodulation-treated eyes were evaluated as either low responders (LRs) (<5 letters at M1) or high responders (HRs) (≥5 letters at M1) to determine the duration of benefit and if PBM treatment benefits were associated with stage of disease. The HR and LR groups were defined as a post hoc analysis. Responder status is determined for each eye, rather than for each subject. At each visit, within each group (HRs and LRs), a paired *t*-test and a Wilcoxon signed-rank test of BCVA change from baseline were performed. For the course of 1 year, statistically significant benefits (*P* < 0.05) were seen for the HR group at M1, M2, M3, M7, and M9 (but not at visits M6 and M12), which were immediately before retreatment and at the conclusion of the study at 12 months. The HR mean BCVA benefit immediately after PBM was 8.0 letters at M1 and 6.0 letters at M7 from BL. The LR eye group did not show any significant benefits over the course of the study.

These two groups were then analyzed for AREDS stage and for foveola involving GA. Approximately 91.3% of HR eyes were either AREDS Category 3 with drusen only or noncentral 1 mm involving GA or AREDS Category 4 with GA, involving the central 1 mm, but still sparing the foveola. By contrast, the LR eyes were primarily AREDS Category 4 (11 of 12), wherein 83.3% (10 of 12) had foveola involving GA.

Photobiomodulation-treated eyes were further stratified by their BCVA-equivalent Snellen score at BL. Three different groups were created and compared. They were eyes with Snellen equivalent scores of 20/200 (ETDRS BCVA letter score of 50) or greater, Snellen 20/100 (ETDRS BCVA letter score of 65) or greater, and Snellen 20/80 set (ETDRS BCVA letter score of 70) or greater. The goal was to further define the treatment response in the different patient groups and to optimize inclusion/exclusion criteria for the future clinical studies by evaluating HRs and LRs by BL vision measurements. Of the LR eyes, 41.7% were eliminated from the study population when the Snellen cutoff was reduced to 20/100. The LR eyes were further reduced to 50% when the Snellen cutoff was further reduced to 20/80. The HR population was only reduced to 91.7% at 20/80. The Snellen population comparison suggests eyes that most significantly respond to PBM treatment are eyes with remaining good baseline vision at Snellen equivalents of 20/100 or better.

### Contrast Sensitivity Assessment

**Fig. 6.**
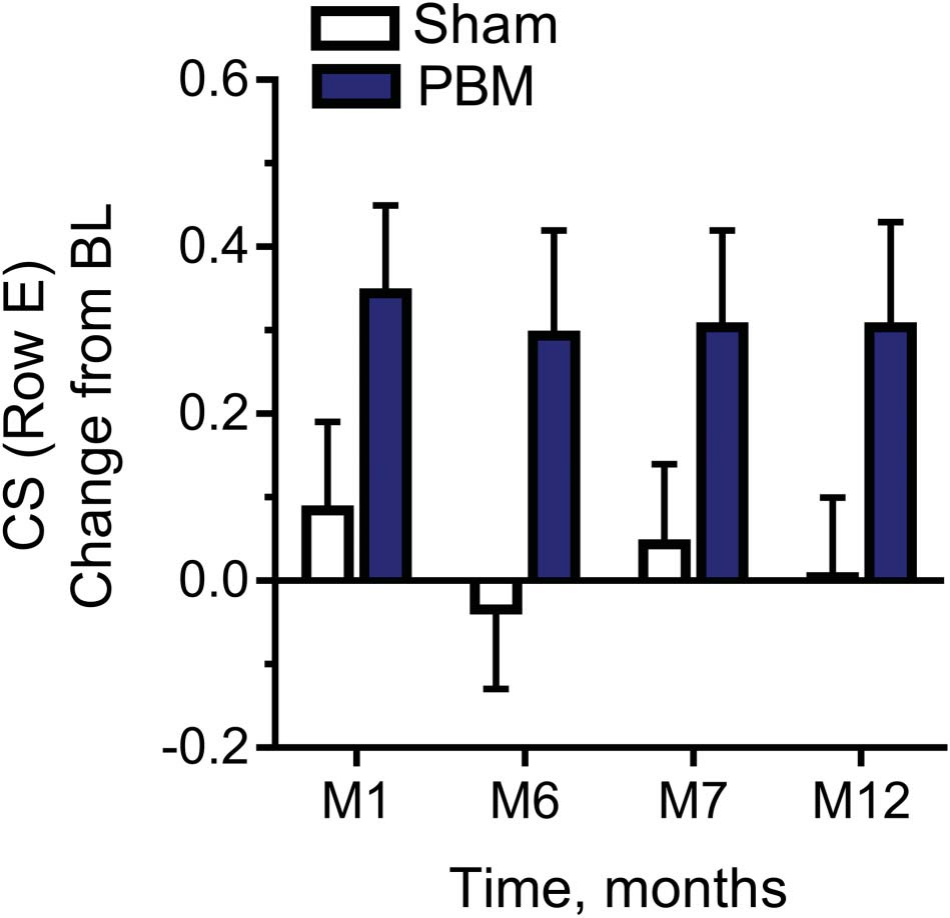
The effect of PBM on contrast sensitivity (Level E, 18.0 CPD) in LogCS change from baseline. Photobiomodulation-treated subjects showed significant improvement in CS at Level E (18 cycles/degree) out to 12 months after treatment, Wilcoxon signed-rank test, *P* < 0.05.

Photobiomodulation-treated eyes showed statistically significant (Wilcoxon signed-rank test, *P* < 0.05) improvement in CS at Level E (18 cycles/degree) out to 12 months after treatment (Figure 6). The increase in CS at M1 was 0.35 + 0.1 and was maintained (0.30 + 0.11) at M12. The Level D (12 cycles/degree) CS data for the PBM-treated group showed a positive trend in benefits over the first 6 months from BL, but the results were not statistically significant (Wilcoxon signed-rank test, *P* = 0.45). The Level B (3 cycles/degree) CS was significant at 12 months between the PBM-treated and sham groups, *P* = 0.026, but not significant at any other time point. Levels A (1.5 cycles/degree) and C (6 cycles/degree) CS data for the PBM-treated group versus sham-treated group were not statistically significant (Wilcoxon signed-rank test, *P* > 0.05).

### Microperimetry Assessment

No statistically significant reduction in bicurve ellipse area measure fixation stability (FS) was observed between PBM- or sham-treated subjects, LME model analysis (*P* > 0.05) at M1 or M12. However, the BL FS levels were higher in the PBM-treatment group, and an improvement in FS after PBM at M1 was seen from 4.4 ± 1.6 to 2.6 ± 0.6 degrees^2^ in the PBM-treatment group. Subsequently, FS values increased in both the sham-treatment and PBM-treatment group over time to reach levels of 4 to 6 before retreatment at M6. For the PBM group, FS at M6 was 6.5 ± 4.0. After the PBM retreatment at M7, the FS values again improved to a mean of 2.3 ± 0.7 degrees,^2^ whereas the sham-treatment group did not respond (LME, *P* = 0.0041).

### Quality of Life Assessment (VFQ-25)

The PBM-treated subjects demonstrated a statistically significant improvement in the QoL composite score at M3 (*P* = 0.003), M7 (*P* = 0.015), and M9 (*P* = 0.003) (Wilcoxon signed-rank test) and select questions related to activities of daily living at M3, M7, and M9. The sham group did not demonstrate a statistically significant improvement (Wilcoxon signed-rank test, *P* > 0.05) in any assessment.

### Anatomical Assessments

**Fig. 7.**
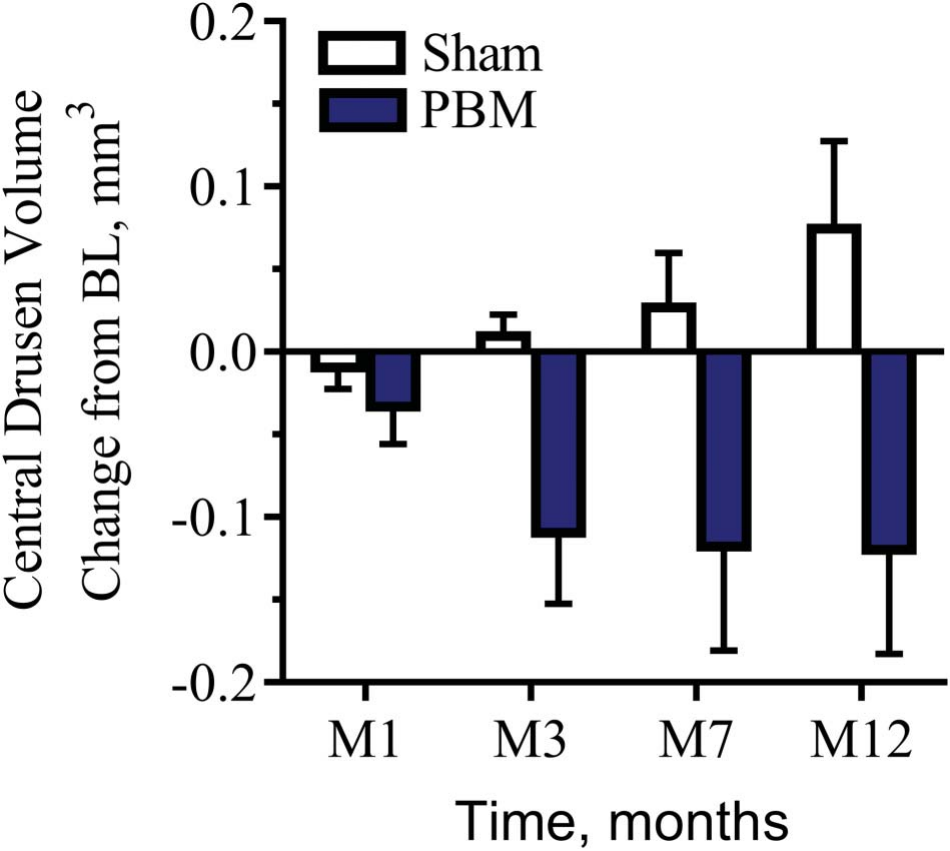
The effect of PBM on drusen volume (mm^3^) over time. Drusen volume increased over time in 100% of the sham-treated subjects. By contrast, 70% of all PBM-treated subjects showed a reduction in drusen. A statistically significant reduction in drusen volume at M12 (LME, *P* = 0.05) was observed in PBM-treated subjects versus the sham-treated subjects.

**Fig. 8.**
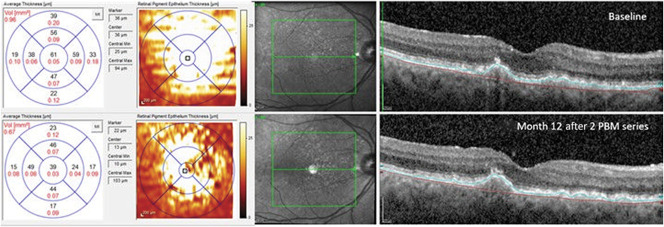
Representative example of anatomical improvement in a PBM-treated eye. This eye was categorized as AREDS 3. Baseline (top) imaging illustrates drusen volume of 0.78 mm^3^ with a mean central 1-mm drusen thickness of 165 *µ*m. Black numbers indicate the mean thickness of each ETDRS subgrid, and red numbers indicate the corresponding volume (mm^3^). The maps on the left-hand side depict the color-coded drusen thickness map. Month 12 (bottom) imaging illustrates an overall reduction in volume (0.41 mm^3^) and mean central thickness (18 *µ*m) after PBM treatment.

Drusen volume increased over time in 100% of the sham-treated subjects. By contrast, 70% of the PBM-treated subjects showed a decrease in drusen volume. A statistically significant reduction in drusen volume at M12 (LME, *P* = 0.05) was observed in PBM-treated subjects versus the sham-treated subjects (Figure 7). No statistically significant difference in reduction in the mean central 1-mm drusen thickness was observed in PBM-treated subjects versus sham-treated subjects at M12 (LME, *P* = 0.18) (Figure 8). Central 1-mm drusen thickness decreased in all eyes, PBM versus sham, and was significant at M7 (LME, *P* = 0.03). No difference in terms of GA lesion growth in the PBM-treated subjects compared with the sham-treated subjects after treatment at 12 months was recorded (LME, *P* > 0.05). No statistically significant change in retinal volume or central retinal thickness was observed in the PBM- and sham-treated groups.

### Safety Assessment

A total of 21 adverse events (AE) were reported during the study (Table 4). In the sham group, 5 AEs were reported by 4 subjects compared with 16 AEs reported by seven subjects in the PBM group. One subject in the PBM group had an eye convert to wet AMD approximately 20 days after the baseline visit. None of the AEs were considered by the investigator to be related to the treatment.

**Table 4.**
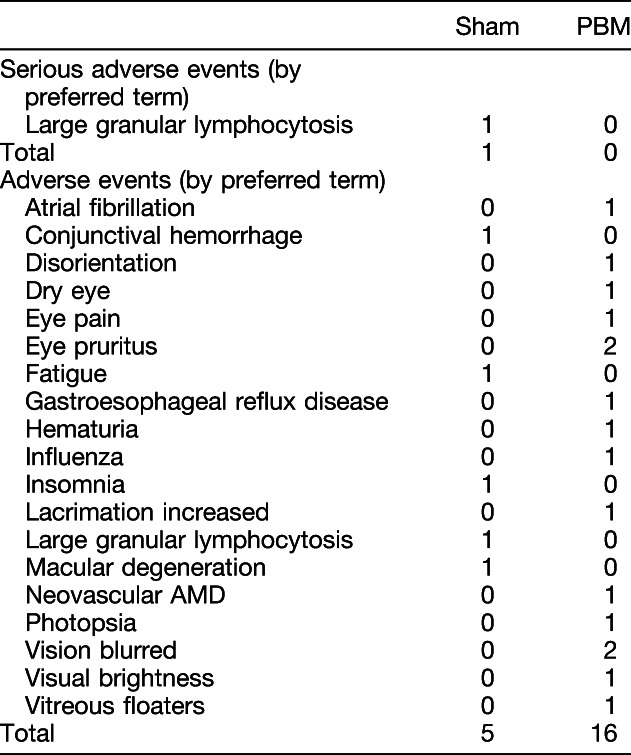
Adverse Events

## Discussion

The LIGHTSITE I study was the first double-masked, randomized, sham-controlled, parallel group study to evaluate PBM in subjects with dry AMD. The results from the study illustrate positive benefits after PBM treatment in both clinical and anatomical outcomes in subjects with dry AMD. These findings corroborate and extend previous reports using the same three wavelengths as delivered in the Valeda Light Delivery System,^23,24^ demonstrating clinical improvement in patients with dry AMD after PBM therapy.

Significant improvements in BCVA and CS were noted at various time points after treatment with PBM throughout the 12-month study. Overall, a mean increase of 4 letters in BCVA was observed at M1 immediately after the first series of treatments. This was followed by a gradual decline in BCVA up to the 6-month mark where the second PBM series was set to take place. The second PBM treatment series immediately improved BCVA letter scores by approximately 4 letters, which similarly declined back to BL levels by M12. These data suggest that PBM efficacy will need to be maintained through repeated 4-month to 6-month retreatment intervals to provide continuous benefits. The molecular underpinnings that drive the effectiveness of PBM make it unlikely that solitary treatment sessions would demonstrate continuous effects. Photobiomodulation dosing protocols commonly use repetitive maintenance doses, and repeated PBM treatments have been suggested by other investigators to stabilize the initial improvements seen in BCVA in other ocular disease states.^22^

Overall, 50% of the PBM-treated subjects showed ≥5 letters improvement after the first series of treatment, and 46% of PBM-treated subjects showed ≥5 letters improvement after the second series of treatments. When looking at letter gain in this subject cohort, a total improvement of 8 letters was observed after the initial treatment series. This subject-specific increase in BCVA letter gain after PBM treatment warrants further investigation and suggests a potential influence of individual disease pathology on the efficacy of PBM treatment. Further exploration of the pathological profile of the enrolled subjects showed that most subjects were classified as AREDS Category 4 with central GA including foveola involvement. Geographic atrophy with foveola involvement was observed in 67.4% of the subjects. Stratifying eyes by BCVA outcomes into those who were HRs (i.e., ≥5 letters improvement after the first series of treatment at M1, HR) demonstrated that high responding eyes were earlier in the disease stage. In the HR group, 66.7% were AREDS Category 3 and 75% had no GA. Most telling was that 92% of the subjects in the HR group had no GA with central foveola involvement. In contrast, in the PBM-treated LR group (i.e., <5 letters of improvement at M1, LR), 83.3% were AREDS Category 4 with GA and central foveola involvement. As disease progression occurs, increased damage and tissue loss are observed limiting the viable retinal tissue that serves as a necessary substrate for PBM activity. Therefore, these findings show that subjects with dry AMD in earlier stages of the disease are more likely to respond better to PBM compared to subjects with more advanced disease and extensive central tissue loss. The high number of subjects with advanced stage AMD contributed to the reduced overall benefits seen in BCVA letter score in the intent-to-treat group analysis.

In addition to BCVA, assessments of CS and microperimetry are suggested to be sensitive parameters of visual function and are impaired at an earlier disease stage. A significant improvement in CS at Level E (18 cycles/degree) was observed immediately after PBM treatment extending to M12. A trend was also noted at Level D (12 cycles/degree) over the first 6 months from BL. Improvements in high performing CS subjects support beneficial changes in visual function, regardless of severity. Significant improvements in FS (microperimetry) were also observed. The combined efficacy of PBM to improve aspects of BCVA, CS, and microperimetry supports the utility of PBM on multiple visual function endpoints.

Functional endpoints such as BCVA and CS are standardized clinical outcome measures for the assessment of disease severity, progression, and response to treatment.^28^ In AMD, improvements in BCVA have become the gold standard for the assessment of efficacy of new treatment options. This is largely attributed to clinical trials surrounding wet AMD where pharmaceutical interventions are used to recover significant acute vision loss through inhibition of neovascularization. In the earlier stages of AMD and also in GA with foveal sparing, the extent of visual dysfunction may remain stable or slowly decline over years without rapid vision loss. Therefore, any visual gain in this patient population, which has not experienced rapid profound vision loss due to CNV, should be considered clinically relevant.

The AREDS data show that about one-third of patients have center-involving GA at the time of initial GA diagnosis. For the remaining two-thirds, there was a median time to progression from foveal sparing GA to central GA of 2 years. Visual acuity is often moderately decreased before the development of central GA, and for those who do not develop CNV, vision is expected to decline an additional 22 letters on average over the next 5 years as soon as the foveal center is involved. That is an equivalent to a loss of approximately 4 letters per year. Eyes that develop subsequent CNV have an even worse prognosis.^29^ A recent study on dry AMD evaluated disease burden and progression in a real-world setting among patients from the United Kingdom with bilateral GA secondary to AMD.^30^ Of the 523 patients who had visual acuity follow-up and a level of visual acuity in their better-seeing eye that would have placed them in a category of eligible to drive at baseline, 349 (67%) became ineligible to drive with a median time to progression of 1.6 years. In the worse-seeing eye, mean visual acuity decreased over 2 years and continued to decline over 60 months. Mean loss of ETDRS letters from baseline was 2.0 letters at Month 12, 6.1 letters at Month 24, and 10.9 letters at Month 60. Over this same timeframe, the better-seeing eye exhibited a steeper trajectory of visual acuity loss; 5.7 letters, 12.4 letters, and 22.6 letters by months 12, 24, and 60, respectively. Clinically meaningful vision loss occurred in both the worse-seeing eye (6.1 letters) and the better-seeing eye (12.4 letters), and the latter rate was twice as rapid compared with the worse-seeing eye. Therefore, we pose that the improvements in BCVA observed in this study are of significance and clinically relevant to this patient population afflicted by the earlier form of AMD.

The results from the LIGHTSITE I study also revealed improvements in anatomical features such as drusen volume and thickness. These findings are consistent with other reports showing similar effects in subjects with AMD after PBM.^23^ Early and intermediate AMD is characterized by the thickening of the Bruch membrane due to the accumulation of lipid and proteins, which form sub-RPE deposits called drusen. Increase of amount of drusen is correlated to disease progression and a risk factor for the development of late complications of AMD including GA, CNV, and subsequent central vision loss. Previous studies report that the rate of progression to advanced AMD (CNV and GA over 5 years) is 1.3% with many small or few medium drusen, 18% if many medium or any large drusen (AREDS, Category 3), and 43% if unilateral advanced AMD is present.^31,32^ There are no approved treatments that currently act to improve vision and influence the hallmark pathology of the disease, so the reductions in these key features of dry AMD are of clinical interest. Sham-treated subjects showed an increase in all eyes in drusen volume throughout the study, whereas 70% of PBM-treated eyes showed a reduction in drusen volume. Improvements in these anatomical features may be correlated to delays in disease progression. However, it should be noted that long-term evidence is needed to correlate drusen reduction with changes in disease progression. Historically, subthreshold laser therapy to reduce drusen has not demonstrated improvements in clinical outcomes.^33^ The Complications of Age-Related Macular Degeneration Prevention Trial was conducted at 22 clinical centers involving 1,052 participants. Participants were observed for at least 5 years after laser treatment. The results of this study provide no evidence of a clinically significant beneficial or harmful effect of preventive laser treatment in eyes with bilateral large drusen at high risk of progression to late AMD. A recent meta-analysis on 11 studies randomizing 2,159 patients showed that laser treatment is capable to reduce drusen volume (OR 9.16), but fails to reduce the incidence of CNV (OR 1.07) or the loss of three or more lines (OR 0.99) at a 2-year follow up.^34^

The Complications of AMD Prevention trial, as well as the meta-analysis, looked at laser and sub-threshold laser, a different technique than PBM treatment, where spot thermal laser applications were delivered to the retinal tissue.^33,34^ In the Complications of Age-Related Macular Degeneration Prevention trial, the initial laser treatment protocol specified 60 barely visible burns applied to specific areas of the retina. At 12 months, eyes assigned to treatment that had sufficient drusen remaining were retreated. The key difference is the use of PBM to stimulate retinal cellular function and not the removal of drusen through cellular repair mechanisms that occur after laser damage to the tissue (i.e., subthreshold). Photobiomodulation has very defined cellular benefits that have been established in many animal models. While differing treatment modalities, PBM will require long-term follow-up to establish that reductions in drusen are correlated to slowing of disease progression. Clinical benefit may not correlate to drusen reduction, and in the current study, the clinical outcomes were seen before significant reductions in drusen. However, drusen pathology leads to further disease progression, and drusen reduction may reflect a shift from deposition to removal as cellular improvements are seen after PBM.

Progressive vision loss is accompanied by a diminished QoL in patients with a diagnosis of AMD. A validated patient questionnaire (VFQ-25) was used to capture subject-reported improvement in QoL measures. Photobiomodulation treatment provided a statistically significant benefit over time, which was consistent with other quantitative clinical outcome measures. Activities of daily living scores were improved after PBM treatment. This improvement is significant in its potential to provide relief to patients. This relief may be measureable in regards to independence and lifestyle limitations which threaten patients with AMD.

A limited number of AEs were reported throughout the study demonstrating a favorable safety profile of the treatment. A total of 21 AEs were reported during the study by four sham subjects and seven PBM subjects. One eye converted to wet AMD in the PBM-treated group within 1 month of the study. The subject was treated with intravitreal anti–vascular endothelial growth factor injections in the respective eye and followed with no further complications. Photobiomodulation treatment was continued for the duration of the study in the remaining dry AMD eye. The dry AMD eye in this subject gained 22 letters by M12 after PBM treatments. The rate of conversion from dry to wet in the current 1-year study was 1 of the 24 PBM-treated eyes for an incident rate of 4.2% or 1 of 46 eyes included in total for an overall incident rate of 2.2%. The published rate of progression to CNV was recently reported as 7.4% per patient-year.^30^ None of the ocular AEs were considered related to the device by the principal investigator and common for the type of disease treated.

There were various limitations to the study inherent to the pilot nature, which include the small sample size and single-center study. Future studies will evaluate increased numbers of patients across multiple clinical sites furthering the safety and efficacy data for PBM treatment in dry AMD. This study enrolled a large number of advanced stage patients. On stratification, a decreased effect of PBM in this cohort of patients was observed reducing the overall effect of PBM in all subjects.

The LIGHTSITE I study suggests the utility of PBM treatment for dry AMD. Clinically significant improvements after PBM treatment were observed in BCVA and CS. Improvements in clinical outcomes after PBM were more robustly seen in subjects with earlier stage disease. In addition, improvements in microperimetry and anatomical outcomes such as drusen volume and drusen thickness were observed. No device-related adverse events were reported demonstrating a favorable safety profile of PBM in dry AMD. These findings support previous reports and demonstrate the potential utility of PBM in subjects with dry AMD.

In conclusion, LIGHTSITE I was an exploratory pilot study that suggested multiple clinical and anatomical benefits after PBM treatment in subjects with dry AMD. The clinical data support preclinical and previous investigator-led clinical studies but herein established in a prospective, randomized, sham-controlled study on how to apply a multiwavelength PBM treatment and gave insight into what patients to target for best clinical results. Further work is planned with multicenter trials with the Valeda Light Delivery System. Most importantly, the LIGHTSITE I results may pave the way for a new treatment approach expanding the field of PBM into the ocular world to combat a debilitating disease with limited patient options. From a clinician standpoint, the treatment may provide an option to their patients to address the disease early, improve visual outcomes, and potentially slow the progression of the disease. We know from experience in other fields that addressing disease early may have the most impact on patient QoL, health care costs, and create the awareness for vision loss prevention. As with most early studies, LIGHTSITE I created additional questions and fostered new ideas that will expand clinical research with PBM, which will provide further insight into best practices for PBM in fighting ocular disease.
